# A systematic review of the literature on ethical aspects of transitional care between child- and adult-orientated health services

**DOI:** 10.1186/s12910-018-0276-3

**Published:** 2018-07-18

**Authors:** Moli Paul, Lesley O’Hara, Priya Tah, Cathy Street, Athanasios Maras, Diane Purper Ouakil, Paramala Santosh, Giulia Signorini, Swaran Preet Singh, Helena Tuomainen, Fiona McNicholas, the MILESTONE Consortium, Swaran Singh, Helena Tuomainen, Jason Madan, Jane Warwick, Cathy Street, Dieter Wolke, Moli Paul, Priya Tah, Rebecca Appleton, Alastair Canaway, James Griffin, Giovanni de Girolamo, Giulia Signorini, Paramala Santosh, Ilyas Sagar-Ouriaghli, Natalie Heaney, Federico Fiori, Diane Purper-Ouakil, Frédérick Russet, Virginie Maurice, Véronique Humbertclaude, Athanasios Maras, Larissa van Bodegom, Mathilde Overbeek, Esther Kooymans, Ulrike Schulze, Melanie Saam, Ulrike Breuninger, Sabine Tremmery, Gaëlle Hendrickx, Fiona McNicholas, Aleksandra Gronostaj, Tomislav Franić, Nikolina Davidović, Frank Verhulst, Gwen Dieleman, Suzanne Gerritsen, Kate Lievesley, Amanda Tuffrey, Anna Wilson, Charlotte Gatherer, Leanne Walker

**Affiliations:** 1 Warwick Medical School, University of Warwick, Coventry, CV4 7AL UK; 2 Stratford CAMHS, Coventry and Warwickshire Partnership Trust, Stratford Healthcare, Arden St, Stratford upon Avon, CV34 6NQ UK; 3 Saint John of God Research Foundation clg, Stillorgan, Co. Dublin, Republic of Ireland; 4 Yulius Academie, Centrum voor Wetenschappelijk Onderzoek, Innovatie en Opleidingen, Dennenhout 1, 2994 GC Barendrecht, Netherlands; 5 CHU Montpellier, Médecine Psychologique de l’Enfant et de l’Adolescent (MPEA1), Hôpital Saint Eloi, 80 avenue Augustin Fliche, 34295 Montpellier Cedex 5, France; 6 Department of Child and Adolescent Psychiatry, Institute of Psychiatry, Psychology and Neuroscience, King’s College London, 16 De Crespigny Park, London, SE5 8AF UK; 7 Psychiatric Epidemiology and Evaluation Unit, Saint John of God Clinical Research Center, Via Pilastroni 4, 25125 Brescia, Italy; 8 Lucena Clinic Rathgar , Dublin 6, Republic of Ireland; 9 University College Dublin, Belfield, Dublin 4, Republic of Ireland; 10 Our Lady’s Children Hospital Crumlin, Dublin 12, Republic of Ireland

**Keywords:** Health, Transition, Care continuity, Adolescence, Young adulthood, Ethics, Systematic review

## Abstract

**Background:**

Healthcare policy and academic literature have promoted improving the transitional care of young people leaving child and adolescent mental health services (CAMHS). Despite the availability of guidance on good practice, there seems to be no readily accessible, coherent ethical analysis of transition. The ethical principles of non-maleficence, beneficence, justice and respect for autonomy can be used to justify the need for further enquiry into the ethical pros and cons of this drive to improve transitional care. The objective of this systematic review was therefore to systematically search for existing ethical literature on child- to adult-orientated health service transitions and to critically appraise and collate the literature, whether empirical or normative.

**Methods:**

A wide range of bioethics, biomedical and legal databases, grey literature and bioethics journals were searched. Ancestral and forward searches of identified papers were undertaken. Key words related to transition, adolescence and young adulthood, ethics, law and health. The timeframe was January 2000 to at least March 2016. Titles, abstracts and, where necessary, full articles were screened and duplicates removed. All included articles were critically appraised and a narrative synthesis produced.

**Results:**

Eighty two thousand four hundred eighty one titles were screened, from which 96 abstracts were checked. Forty seven full documents were scrutinised, leading to inclusion of two papers. Ancestral and forward searches yielded four further articles. In total, one commentary, three qualitative empirical studies and two clinical ethics papers were found. All focused on young people with complex care needs and disabilities. The three empirical papers had methodological flaws. The two ethical papers were written from a clinical ethics context rather than using a bioethical format. No literature identified specifically addressed the ethical challenges of balancing the delivery of transitional care to those who need it and the risk of pathologizing transient and self-limited distress and dysfunction, which may be normal during adolescence.

**Conclusions:**

There is very little research on ethical aspects of transitional care. Most existing studies come from services for young people with complex care needs and disabilities. There is much scope for improvement in the amount and quality of empirical research and ethical analysis in this area.

**Electronic supplementary material:**

The online version of this article (10.1186/s12910-018-0276-3) contains supplementary material, which is available to authorized users.

## Background

For over a decade, healthcare policy [1–3] and international academic literature [4–7] have promoted improvement of the quality of transitional healthcare for young people who transfer from child and adolescent mental health services (CAMHS) to adult mental health services (AMHS). They have also raised concern about those young people with ongoing mental health needs who ‘fall through the gap’ [6, 8–12]. Despite the availability of guidance on good practice [1, 3], there seems to be no readily accessible, coherent ethical analysis of this type of transition. This study is also necessary in order to maintain an enquiring ethical stance in the face of an ever increasing body of clinical and health services research that *works on the assumption* that promoting good quality transition and achieving a transfer of care from child to adult services is *good*.

Using Beauchamp and Childress‘commonly used Four Principles approach [13], which balances beneficence (maximizing benefit), non-maleficence (avoiding or minimizing harm), respect for autonomy (respecting decision-making capacities of autonomous people and helping them make informed choices) and justice (fairly distributing benefits, risks and costs), we can illustrate the need to expand the ethical analysis of transitional care. On the one hand, approximately one in five adolescents have a mental disorder, which predicts having homotypic and heterotypic disorders in adulthood [14]. The transition boundary between CAMHS and AMHS is generally when the young person is between 16 and 18 years of age. Transfer to AMHS would arguably provide the benefit of appropriate mental health care and minimize harm from untreated disorder. Firm guidance on making transition developmentally-appropriate and treating young people as equal partners in the process [3] could be seen as promoting and respecting autonomy. The current disjunction between CAMHS and AMHS creates systemic weaknesses in healthcare just when severe mental illnesses tend to emerge, unfairly undermining continuity of care just when it is needed most [9, 15].

On the other hand, despite the policy and good practice guidance, research indicates that CAMHS to AMHS transitional care remains at best patchy [16], there is often a policy/practice gap [11, 17], and the evidence base about ‘what works’ is weak [18, 19]. All these factors undermine arguments that benefit, reduction of harm and fair provision of services occur in the real world. Young people sometimes choose not to attend AMHS, hence always promoting transition could undermine respect for autonomy [10, 20]. The possible harm of pathologizing transient and self-limited distress and dysfunction, which may be normal during adolescence, had been highlighted by reviewers of previous papers [11, 17] and was discussed within the research group. It may also be harmful to recommend transfer to a service that may not meet the needs of young people, just because that is the structural service option available.

Through undertaking previous studies [12, 17] and systematic reviews [18, 21] we were aware that literature on ethical and legal aspects of transitional care was difficult to find. Our objectives therefore were to systematically search for, critically appraise and collate the literature on ethical aspects of transitional care between child-orientated and adult-orientated health services in general. The research questions were:

1. What types of literature exist on ethical/legal aspects of transitional care?
2. Does empirical research exist on ethical aspects of transitional care?
3. What is the quality of any empirical research?
4. What is the quality of any ethical/legal analysis?
5. What are the ethical/legal challenges of ensuring delivery of transitional care to those who need it most against the risk of pathologizing transient and self-limited distress and dysfunction, which may be normal during adolescence?

Traditionally, literature reviews in bioethics have captured key issues instead of comprehensively identifying and analyzing all relevant literature [22]. McDougall (2014) [22] identifies three types of bioethics systematic review, based on whether or not the research question is ethical or empirical and whether the ethical literature being reviewed is normative (to establish basic ethical principles or standards) or empirical. This ethics systematic review started from a position of not knowing what types of evidence we would find – empirical or normative – hence our approach has been hybrid, i.e. we undertook a systematic review of both the empirical and normative bioethics literature.

## Method

### Search strategy

The overall search strategy was agreed by MP/LO/PT/CS/FM but LO/PT/MP undertook searches of specific databases and journals. The following bioethics and biomedical databases were searched: Project MUSE, PubMed, PsycINFO, CINAHL, EMBASE and Web of Science (previously known as Web of Knowledge). Two legal databases, Lexis Library and Westlaw UK, were also searched as some jurisprudential arguments indicate that ethics is an integral part of the law [23]. Ancillary searches included a systematic search of the Grey literature, including ProQuest Dissertations and Theses and Google Scholar.

We also undertook ancestral searches (references of any reviews and papers identified through searching databases and the Grey literature); forward searches for articles citing key papers or other documents; and searches of specific specialist journals published since January 2000. The journals searched were Ethics (previously known as International Journal of Ethics and Journal of Ethics, <April 2016); Ethics and Behaviour (<May 2016); Ethical Theory and Moral Practice, Journal of Medical Ethics (and supplements), Philosophy Ethics and Humanities in Medicine (from first volume in March) and the Hastings Centre Report (all<June 2016); and Philosophy Psychiatry and Psychology (<September 2015).

### Search terms

Key words and their truncations and relevant database-specific subject headings and [MeSH terms] were used, targeting all four of the following subject areas on an AND/OR basis:

- Transition: including transition*; interface*; transfer; Care continuity; Continuum of care; Care continuum; Patient care continuity [Transition to adult care; Continuity of patient care; Referral and consultation]; becom* adult; “CAMHS to AMHS”; “child and adolescent mental health services to adult mental health services”
- Age: including young; youth*; teen*; adolesce*; young adult; youth; transition age youth; emerging adult*, or age-group criteria (e.g. “adolescence” (13-17 years AND “young adulthood” (18-29) in Medline)
- Health: including youth services, adolescent health services; child health service*; child and adolescent mental health service*,
- Ethics/law: including Ethic*, Moral, Legal, law [Ethics; Ethics, professional; Ethics consultation; Morals; Jurisprudence]

Search terms were combined in various ways depending on the functionality of the search engine, e.g. where multiple terms could be added on an OR basis for each category, they were. When advanced search options were not complex enough, more limited searches were done and collated. Pragmatic approaches were taken to broaden the search when initial searches produced no results.

### Inclusion/exclusion criteria

No exclusions were made on language grounds. Given transitions-related literature, including terminology, started in the early 2000s, the search included documents from January 2000 to at least March 2016. Papers on the nature of health and illness [24] and mental illness or mental disorders [25, 26] were excluded.

### Data extraction and mapping

MP/LO/PT checked titles and abstracts to assess whether articles fulfilled inclusion/exclusion criteria and removed duplicates. Any doubts about meeting inclusion criteria were resolved through retrieval of full articles and team discussion (MP/LO/PT/CS/FM). Full versions of the yield were read by a researcher (MP, LO or PT), who checked against inclusion and exclusion criteria, to identify systematic reviews, reviews, ethical analyses, legal analyses and primary research. Uncertainty about inclusion was resolved through review of each paper by two others from the reviewing group (MP/LO/PT). Ancestral searches of the references of reviews and identified documents were undertaken (MP/LO/PT) and repeated recurrently. Experts in the field were also consulted about additional reviews and primary research. A PRISMA chart [27] was planned however there was no way of collating searches to remove duplicates as the two research sites did not share common bibliography software. Findings were therefore tabulated (Table 1), with duplicates removed at a later stage.

**Table 1:** Sources of literature and reasons for discarding search findings

Database	Search timeframe 01.01.00 to	Titles viewed	Abstracts viewed	Papers viewed	Papers included	Examples of what excluded publications considered
Project MUSE	07.03.16	132	8	1	0	Transition to adulthood; other types of transition such as marriage and cohabitation, child bearing, employment, school to employment, high school to college, immigration, political (democracy); other interfaces, e.g. between psychiatry, psychology and philosophy; and other types of transfer, e.g. transfer of technologies between countries
PubMed	20.03.16	175	6	3	1^30^	The structure of CAMHS in a number of geographical locations; ethical aspects of psychiatric assessment of young people in immigrant detention; transition to end-of-life care; and the process of transition from child to adult healthcare in the context of ASD but without consideration of ethical issues
PsycINFO	20.03.16	70	4	1	0	Moral dilemmas in psychiatry (but not addressing transition), capacity to consent and the transition to end-of-life care
EMBASE	20.03.16	126	4	3	1^31^	A narrative summary of the transition process within the context of chronic illness and a discussion of the ethical aspects of the treatment of chronic conditions which made no reference to the issue of transition
Web of Science	20.03.16	309	16	6	1^30^	systematic reviews of the literature on transition that did not consider the ethical/legal aspects of transition, and articles which considered practical issues regarding transition but did not attend to the ethical/legal issues
CINAHL	20.03.16	55	2	2	0	supporting young people in transition, service design, and the treatment of young people with specific mental illnesses
Lexis Library	21.03.16	4668	6	0	0	records about transitions specific to youth justice and implications of mental and public health policy
Westlaw UK	27.03.16	1325	2	0	0	services specifically for offenders which did not focus on health
Google Scholar advanced search	04.04.16	53,281	28	15	0	Transition between child and adult (including mental health) services, which did not focus on ethical aspects
ProQuest advanced search	30.03.16	22,340	20	16	0	Processes related to and readiness for transition and ethical issues of transition, especially trust, but from hospital to specialist home care rather than child-adult services

### Critical appraisal and synthesis of research

#### Critical appraisal of the empirical bioethics literature

The approach to empirical studies was similar to that used in previous systematic reviews on transitions literature [18, 21]. A Mixed Studies Review methodology was used, including utilization of a data extraction form (see Additional file 1) and a validated critical appraisal tool for empirical studies (see Additional file 2) [28]. The scoring system covers nine components of critical appraisal, including those likely to create bias: abstract and title, introduction and aims, method and data, sampling, data analysis, ethics and bias, findings/results, transferability/generalizability, implications and usefulness. Each study is given a quality score of 1 (very poor), 2 (poor), 3 (fair) or 4 (good) on each component, generating a maximum potential score of 36. The criteria for appraizing “introduction and aims” was amended, as had been done previously [18], in that the ‘fair’ score criteria were changed to include “aim OR objectives OR research questions” rather than requiring more than one.

#### Critical appraisal of the normative bioethics

For the normative ethical and legal literature, we used an assessment tool incorporating four questions: first, does the article address a focused ethics question? Second, are the arguments that support the results of the article valid? Third, what are the results? Fourth, will the results help in practice? [29].

#### Synthesis

As very few articles were identified, a narrative synthesis was used for all papers. This involved providing a summary of the findings (if empirical) or arguments (if ethical) of each paper.

## Results

### Identification of literature

Eighty two thousand four hundred eighty one titles were screened, from which 96 abstracts were checked. Forty seven full documents were scrutinised, leading to the inclusion of two papers [30, 31]. No documents were included from searches of Project MUSE, PsycINFO, CINAHL, Lexis Library or Westlaw UK. PubMed yielded one [30], which was also the only article identified from Web of Science. EMBASE yielded another [31]. Neither Google Scholar nor ProQuest advanced searches were successful (see Table 1). The searches of ethics journals yielded no results. Ancestral and forward searches were most fruitful, yielding four further articles [32–34]. In total only six articles were identified, organized below according to year of publication.

### Bailey et al. (2003) [35]

This paper is a commentary, providing ethical perspectives from three Australian academic nurses, in the context of the transition of young people with disabilities from pediatric to adult services. They argue that relationships between young people with disabilities (and/or their carers) and the pediatricians they have known through long periods of their childhood often entail dependency and paternalism. The paediatrician’s authority is emphasised in these relationships, which then undermines promotion of young people’s well-being and their/carers‘successful negotiation of adult healthcare services.

The ethical argument, supported by government policy, empirical studies, ethics literature and their own experiences, can be understood as follows: Promoting autonomy, which they more or less equate with self-determination, is good because it enhances well-being, maximizes the likelihood of the young person being the main decision-maker about their own healthcare and, as the moral basis of personhood, enhances personal worth and dignity.

The authors acknowledge the rationale for paternalism is to prevent negative outcomes for the young person and/or their carer, especially when the autonomy of the young adult may be limited by their, for instance, intellectual disability. They argue, however, that self-management and autonomy should be promoted, whenever possible, to increase the individual’s self-esteem and self-respect. Acting paternalistically could also create harm by creating distress at leaving pediatric care and reducing the young person‘s (and carers‘) future ability to negotiate adult healthcare systems, thereby undermining health outcomes. They conclude that a formal transition program should be in place to promote young people’s autonomy but do not suggest a method for operationalization. The paper is therefore better at explaining the problem than giving practical solutions (see Table 2 for more detailed critical appraisal).

**Table 2:** Critical appraisal of the normative ethical literature

	Does the article address a focused ethics question?	Are the arguments that support the results of the article valid?	What are the results? What are the conclusions of the paper’s ethical analysis and argument?	Will the results help me in clinical practice?
Bailey et al. (2003) (33)	Yes.	In part: This paper is a commentary rather than a formal ethical analysis.	Obstacles to effective transition are detailed and authors conclude that a formal transition program should be in place to promote autonomy and self-determination in the young person and the carer/family members.	A little: Although statements about developing a more effective transition program are made, how this should be achieved is not operationalized.
Kaufman et al. (2010) (31)	Yes.	In Part: The authors drew on arguments, mixing differing ethical issues, theories (starting with a duties-based approach and ending with consequentialist outcomes) and principals.	A number of ethical issues are identified, including dignity and respect, trust, graduated capacity, promoting autonomy, duties (including beneficence, non-maleficence, truth-telling, advocacy and providing developmentally-appropriate care)	Yes: Numerous recommendations are made about how to act on the identified ethical issues.
Racine et al. (2014) (29)	Yes	Yes	Four ethical considerations are identified: autonomy in transition; youth-provider relationships; development of ethics in transition programs; and the ethical challenges during transition among young people with neurodevelopmental disabilities.	Yes. A paradigm of personalized transition is elucidated, including suggestions on how to overcome barriers.

### Kaufman et al. (2010) [33]

This clinical ethics paper by three Canadian academics, a bioethicist, a pediatric nurse and a pediatrician, explores provision of transitional care. The context is the transition of young people with special healthcare needs from pediatric to adult services.

The authors’ perspective is that “ethics in health care is about preserving, promoting and ensuring dignity and respect for patients” (p454) and that children have human rights to develop physically and mentally, with freedom and dignity, as enshrined in the United Nations Convention on the Rights of the Child [36]. They stress the duties and obligations of healthcare professionals to support young people in developing agency, whilst protecting them on their path to full capacity. They argue that the law creates an arbitrary cut off below which young people are considered unable to reach and maintain decisions; this stops them gradually developing responsibility as consumers of healthcare. Yet, this responsibility is required when they start to use adult services.

Five clinical case studies of young people with congenital anaemia, severe cardiac disease, systemic lupus erythematosus, haemoglobinopathy and autism are used as illustrations. In one case, a provider took on a ‘protector’ role with a patient, perhaps stereotypically perceived as vulnerable, and misrepresented transition options to assuage distress (for patient and provider), risking the patient losing trust in the future and failing to be prepared adequately for transition. In another, the transitioning patient had been told for years by pediatric clinicians how poor the adult services were compared with pediatric services, resulting in fear and uncertainty about future relationships and care. The third involved a mother, who had always spoken for her child in pediatric services, being asked to wait outside the consulting room in the adult service. Fourth, a 22 year old still attending a children‘s clinic, due to the lack of specialist adult services, protested when her height was still measured at each visit, despite having reached her adult height five years previously. In the last case, a young person with autism had sensory sensitivities that made it hard for him to use busy, general adult services.

Ethical issues identified included: preserving, promoting and ensuring dignity and respect for patients; fostering and supporting trusting relationships between young people (and their parents) with new adult providers by using a graduated system of transfer; recognizing graduated capacity; promoting autonomy and self-management; duties of beneficence and non-maleficence; truth-telling; duty to provide developmentally appropriate care; and pediatric providers‘ duty to advocate for transitioning patients in the adult system. The authors utilise differing ethical theories and principles. They provide detailed advice on what to do to improve ethical, clinical practice and acknowledge that supporting young people in developing agency needs to be considered alongside protecting them when they do not have full capacity (see Table 2).

### Racine et al. (2013) [32]

This qualitative study, from a group of ethicists and clinicians from pediatric and pediatric neurology services in Canada, investigates respect for autonomy in the context of young adults with cerebral palsy. Sampling strategy was not described but semi-structured interviews were conducted with and a short questionnaire administered to 14 individuals, aged 18-25, with a range of levels of communication and physical disability. Interview questions and discussion focused on topics including ethical and social issues encountered in healthcare, such as autonomy, making medical decisions and relationships between participants and healthcare professionals.

Thematic qualitative content analysis was undertaken. Results indicated that all participants explicitly or implicitly suggested that autonomy was valuable. Several, however, said autonomy should be seen alongside competing values (e.g. parents or a spouse acting for their welfare or in their best interests) and in context (e.g. making a decision jointly with parents who have always respected their views). Most participants’ autonomy had been generally promoted in health services, i.e. their decisions, including refusals of treatment, had been respected, although some experienced the opposite in both pediatric and adult settings.

Non-health related issues that impacted on autonomy/independence in health settings were a lack of public transport for disabled people and not having home support at appropriate times, e.g. early enough to help get to an early hospital appointment. This sometimes led to reliance on family for transport, resulting in family presence at appointments. Some participants thought their parents were making the right decisions for them, even within the adult system and delegated their decision-making authority. Others’ autonomy was hindered or thwarted by their parents’ continued presence at medical appointments. Some participants reported that healthcare providers exhibited both enabling and disabling attitudes, in relation to providing information about their condition and treatment options, involving them in healthcare decision-making or making overprotective/enabling recommendations.

The authors conclude that autonomy is highly valued but competes with other goods, such as maintaining relationships with parents and getting practical support from relatives, and other ethical principles, e.g. beneficence. They identify “multiple tension points” (p877) where respect for autonomy may be fragile. These include times when decisional autonomy and physical autonomy, or independence, become coupled: the lack of the latter should not automatically equate with lack of the former. Family can both promote and oppose young people’s individual autonomy. Professionals’ negative assumptions and attitudes can have a considerable impact on young adult patients’ autonomy and independence, leaving them ill-prepared for taking on full decision-making autonomy during the pediatric–adult transition process.

### Larivière-Bastien et al. (2013) [34]

This qualitative study investigates the perspectives of 14 young adults with cerebral palsy on transitioning from pediatric to adult healthcare systems “through the lens of ethics” (p155). From the same Canadian authors who wrote the Racine et al. (2013) [32] paper, it seems to have the same participants: nine had transitioned, one was in the process, three still attended pediatric services and one lived in an area without specific pediatric care.

Semi-structured interviews were undertaken. Thematic content analysis explored key ethical aspects during the transition process, including if and how: individuals feel respected; their values and preferences are developed and integrated; and they are prepared to participate autonomously in decision-making.

Six participants reported some positive experiences, including adequate preparation for themselves and support for their families during transition. They were all satisfied with adult services, which they perceived as more personalized.

Eight types of concerns were identified. Fear and apprehension, before transition, mainly centred on losing easy access to pediatric services. During the transition process, participants reported a lack of cooperation, poor communication and contradictions between pediatric and adult service clinicians. Several participants felt inadequately prepared, supported or informed. Medical files were often not transferred. Adult services provided shorter appointments, fewer adjunctive therapies (e.g. speech and occupational therapy) and less access to other types of professional (e.g. psychiatrists); they were perceived as having less understanding of cerebral palsy and less supportive, e.g. not providing reminders about appointments. After transition, some participants had felt abandoned and others sad at having left the pediatric system.

The ethical analysis linked results to respect for persons and respect for autonomy. Authors suggest that transition programs could dedicate specific attention to ethics and decision-making by identifying core competencies for service users to develop and validate, possibly using patient-reported outcome measures (PROMs). Promoting respect for autonomy was also seen as a way of minimizing harm and coinciding with clinical goals, such as preparation for autonomous decision-making.

### Racine et al. (2014) [31]

This clinical ethics paper summarizes the discussion at a Canadian national conference on the ethical challenges of transition from pediatric to adult health care services for young adults with neurodevelopmental disabilities such as autism spectrum disorders, cerebral palsy and foetal alcohol syndrome. The conference included presentations on relevant research, including two of the above reported papers [32, 34]. Rather than using traditional methods of ethical analysis, authors used an ethical ‘lens’ (p65) to focus on the values and preferences of stakeholders, bringing to attention how general ethical principles are challenged or promoted in transition. A collaborative writing process was used to consolidate the discussion, which revealed four themes:

1. Most transition-related literature accepts the development of autonomy and young people’s participation in decisions about their healthcare in age−/developmentally-appropriate ways as a fundamental goal of good transitional care. Some young people with neurodevelopmental disabilities, however, may value shared decision-making with their parents and/or have personal targets that may not always coincide with practical and decision-making independence. A more personalized set of goals in transitional care is required, so as not to idealize independence or intimate that lack of autonomy, in terms of practical and decision-making independence, equates with a lack of personal responsibility.
2. Healthcare providers can bring about harms or benefits in the process of transition. Harms from sub-optimal transition can include financial costs and a loss of opportunities for the individual and family. Society also loses, if young people with these types of difficulty are impaired in terms of contributing to the diversity of society.
3. Transition programs should have explicit ethical goals, including the formulation of a personalized definition of ‘good transition’ for each young person. Social or contextual aspects that can compound or diminish disability should also be explicitly addressed.
4. A “middle-ground approach” (p67) is required to balance improving transitional care for an individual with neurodevelopmental disabilities and what is good for the larger group of transitioning young people, who have heterogeneous needs. Supplementing core transitional care with tailored modules was proposed. The need for research to evaluate health economic and quality of life outcomes and personalized transition goals was also highlighted.

The paradigm of personalized transition, with its ethical underpinnings and many linked practical suggestions, was the key outcome. Respect for autonomy was said to be valuable to the extent that it is “appropriate and possible” (p67).

### Affdal et al. (2015) [30]

This qualitative study, from a group of French bioethicists and pediatric neurologists considers transition from child to adult health services in the context of young people with epilepsy. The authors aim to analyze transition arrangements and to explore the issue of patient autonomy.

Individual, semi-structured interviews were undertaken with ten doctors from six Parisian hospital pediatric neurology departments. None of the interviewees were working within a multidisciplinary network. The interviews focused on the criteria for transition to adult services, processes of transition (including joint-working between pediatric and adult clinicians along with transfer of medical records) and the role of psychologists and social workers in the process of transition.

The results illustrate that the age of majority is a major criterion for transitioning someone to adult services. Pediatricians felt protective about the young people they have cared for and sometimes struggle to refer them to adult neurologists. Some pediatricians raised transition merely implicitly but others engaged in detailed discussion with the young person and sought their agreement. Most discussion was with parents/carers as virtually none (9/10) of the pediatricians spoke to their adolescent patients directly or alone. In all cases, discussion about transition was initiated by the pediatrician. For the most part, the pediatrician considered the competency of the hospital-based neurologist and their working relationship, as opposed to proximity to the family, when deciding upon a referral route.

The issue of autonomy is mainly addressed in the discussion rather than being evidenced in the results. The extent to which pediatricians really considered the autonomy of their adolescent patients was questioned. An opinion is offered, unsupported by data, that parents too can be very protective, thus further weakening the patient’s autonomy. The practice of adolescent medicine, which includes specific foci on supporting the development of adolescents‘autonomy and the training of doctors to facilitate this, is promoted as one way forward.

### Critical appraisal of empirical studies

The details of the critical appraisal are given in Table 3. Of note is that, for all three, method and data, as well as transferability/generalisability were scored as poor. For all three again, sampling was scored as very poor and ethics and bias as poor or very poor. The latter particularly suffered from few details on confidentiality and consent-seeking for research participation.

**Table 3:** Summary of quality scores for empirical studies (*n* = 3)

	Racine et al., 2013	Larivière-Bastien et al., 2013	Affdal et al., 2015
1. Abstract and title^a^	Good	Fair: Abstract not structured but this could be because of the style of the journal. Key ethical constructs not mentioned	Good
2. Introduction and aims^a^	Good: Reasoned ethical argument and clear aim	Fair: Research questions clear but introduction not a systematic summary of the background literature	Good
3. Method and data^a^	Poor: Qualitative interview and questionnaire mentioned but no subsequent mention of questionnaire. Questionnaires/ interview schedule not included	Poor: Ostensively this is a study about ethics and the researchers have taken an inductive approach but present a deductive structure for their paper, even criticising themselves for generating hypotheses rather than confirming or refuting. Questionnaires/ interview schedule not included	Poor: The method did not fit with the aim of the study. Adolescents were not interviewed regarding their autonomy. No detail of analytic approach given
4. Sampling^a^	Very poor: Description of sample but nothing about appropriate sample size or sampling strategy	Very poor: Description of sample but nothing about appropriate sample size or sampling strategy	Poor: Sampling strategy is absent. Very few participant details are given
5. Data analysis^a^	Fair: Description of method but no respondent validation or triangulation	Fair: Description of method but no respondent validation or triangulation	Very poor: No details of the analytic approach are provided
6. Ethics and bias^a^	Poor: Ethical approval for the study gained. Issues of confidentiality, approach to gaining consent to participate not mentioned although reader referred to more details about methodology in another paper. Relationship between researcher and participants not discussed	Poor: Ethical approval for the study gained but not sure from where. Issues of confidentiality, approach to gaining consent to participate not mentioned although reader referred to more details about methodology in another paper. Relationship between researcher and participants not discussed	Very poor: No reference is made to ethical permissions. Issues of confidentiality, approach to gaining consent to participate not mentioned
7. Findings/results^a^	Good	Good	Fair
8. Transferability/ generalizability^a^^b^	Poor: Poor description of context. Poor score in 4	Poor: Poor description of context. Poor score in 4	Poor: Poor score in 4
9. Implications and usefulness^a^	Fair: Some suggestions for changing practice and transition programs. No implications for further research	Fair: Some suggestions for changing practice. Mentions focus for further research	Fair: No suggestions for future research given
Total (out of 36)	25	23	22

^a^Good = 4, Fair = 3, Poor = 2, Very Poor = 1

^b^In order to score  Good or Fair score for transferability/generalizability, the paper should score Good or Fair for question 4 (Sampling).

## Discussion

### Main findings

One commentary, three empirical studies (all presenting qualitative data) and two clinical ethics papers were found on ethical/legal aspects of transitional care. All papers focussed on the needs of young people with complex care needs and disabilities and four of the six were from Montreal, Canada. No papers were found on ethical/legal aspects of transitional mental health care.

The quality of the three empirical studies was poor. Lack of information on sampling strategies, failure to provide semi-structured interview schedules and address research ethics issues, such as consent to participate in the studies and confidentiality, were ubiquitous. A likely lack of data saturation due to small samples and the impossibility of triangulating findings (as only clinicians or only young people were interviewed) also limited the small evidence base.

The two non-empirical papers were written ‘through the lens of ethics’, from a clinical ethics perspective. ‘The lens‘ focusses on the values and preferences of service users and how ethical principles are promoted or challenged during young people’s transition. It does not use traditional methods of ethical or philosophical analysis [37], e.g. it does not use explicit premises that are examined and challenged. Clinical cases were used to exemplify ethical issues rather than ethical case comparison being used to ethically examine the pros and cons of, for example, promoting autonomy. No thought experiments were used. In general, reasoning from principles was not seen through using logical argument and conceptual analysis. As a result, the ethical worth was one of promoting values espoused by authors, e.g. that clinicians have duties to help young people develop autonomy and to listen to young people’s views.

No identified literature answered the question “What are the ethical/legal challenges of ensuring delivery of transitional care to those who need it most against the risk of pathologizing transient and self-limitieddistress and dysfunction, which may be normal during adolescence?”. It is tempting, both given the paucity of evidence found and the vast amount of literature searched and screened, to provide more in this discussion from papers that did not meet our inclusion criteria - papers that, in our view as authors, investigate issues of relevance to this last research question. To do so, however, would produce a discussion reflecting our own perspectives and values, rather than our findings.

### Could the methodology have been better?

The systematic search strategies used for finding empirical research were thorough and robust. One improvement when searching for argument-based ethics literature could have been to frame our fifth research question in a PICO (population, intervention, comparator and outcome) format [38]. If we were to repeat the systematic review, we would not search the legal databases, given the large numbers of titles identified that ultimately did not provide a yield.

Alternatively, perhaps a systematic review is not the best way to identify or collate relevant ethical issues. McDougall (2015) [39] has proposed that a good bioethics literature review does not have to be systematic – instead it should capture and analyze the key ethical ideas of relevance to the research question, i.e. be a critical interpretive synthesis. Such a synthesis should include the following six features:

1. answering the research question
2. capturing key ideas of relevance to the research question
3. analyzing the literature as a whole
4. generating theory
5. not excluding papers purely on rigid quality assessment criteria, and
6. reporting the search strategy

Our experience in undertaking this systematic search would re-enforce this view as we had to exclude a number of papers that raised relevant issues but did not meet all the inclusion and exclusion criteria.

### Implications for practice

The common underlying ethical themes from identified papers were: the principles of respect for autonomy, beneficence, non-maleficence and justice; promoting autonomy whilst acknowledging internal autonomy-limiting issues, such as limited cognitive capacity or impaired communication skills; promoting autonomy whilst acknowledging external, practical barriers to achieving autonomy, such as poor public transport for disabled people leading to reliance on family; acting in best interests and paternalism; rights to privacy, self-determination and participation in healthcare decision-making with adequate information to make informed decisions; values and preferences of stakeholders; duties of care, including respecting the young person’s values and providing appropriate care and advocacy when needs are not met; the virtues of truth-telling and being trustworthy; dignity, personhood and respect for persons.

Racine et al. (2014) [31] already summarize a number of implications for practice, under their proposed paradigm of personalized transition. Most of their suggestions are around promoting certain ethically good actions and attitudes in order to improve transitional care. They mention balancing individual-centered goals and family- or society-centered goals but there is more scope for promoting the skills required to balance ethical principles. Similar skills should be developed to aid analysis of virtues (e.g. being caring and minimizing distress vs being honest about differences in provision of care when moving to adult services) and theories (e.g. deontological duty of care vs consequentialist maximisation of choice or health outcome).

Overall, personalised care (a healthcare concept that requires ethical unpacking), choice (the young person’s choice, parental choice and professional choice), autonomy (whether present and to be respected or absent and to be promoted) and relationships (between young person and clinician, young person and carer, clinician and carer and between all relevant parties) seem to be over-arching themes in a context full of competing ethical concepts, e.g. respect for autonomy and advocating for young people lacking autonomy. Promoting young people’s participation in decision-making, whether or not they are autonomous, is a ubiquitous aim, but fraught with ethical and practical variability. A unitary approach cannot provide this personalised care as young people at transition boundaries have such wide-ranging circumstances, including differences in cognitive abilities, practical independence, type and impact of health difficuties and significant others (i.e. the values and views of parents or carers and relevant professionals can vary). In clinical practice, it would seem that ethical reasoning should remain tethered to the individual in their context, i.e. be undertaken on a case by case basis.

### Implications for future research (empirical and theoretical)

There is wide scope for future research into ethical aspects of transition in both western and non-western countries, in particular in the area of mental health. This includes empirical, applied clinical ethics research; formal, bioethical scrutiny of relevant issues using traditional tools such as distinguishing facts from values, clarifying the logical form of the argument, analyzing concepts, reasoning from principles and theories, using case comparison or thought experiments [37]; and combined methods such as McDougall’s critical interpretive synthesis [39].

Promoting the development, or advocating for the recognition, of young people’s autonomy differs from respecting only the autonomy of those who have autonomy. Some of the above papers do refer to the need to balance respecting young people’s choices with other ethical goods such as not doing harm or indeed other people’s choices such as their parents’. Some acknowledge that perhaps certain young people may not have autonomy because of their intellectual or physical disabilities. Papers in the future could be more specific about any internal (e.g. cognitive difficulties) or external (e.g. the social and political disabilities of being minors) limitations on the ability of the young people they are thinking about to achieve autonomy [40, 41]; authors could also be more specific about when, in respecting young people’s dignity, they are promoting rights proportionate to an individual’s agency, rights appropriate to any vulnerabilities they may have and preparatory rights that aim to help young people develop agency [40, 41].

Further theoretical ethical analysis could also inform future empirical bioethics research and vice versa. Borry et al. (2004) [42] note how empirical research can inform three key steps in ethical reflection: description of the moral question, assessment of the moral question and the evaluation of the decision-making.

## Conclusion

There is very little research on ethical and legal aspects of transitional care. Most of the existing few studies come from services for young people with complex care needs and disabilities. There is much scope for improvement in the quality of empirical research and ethical analysis in this area.

## Additional files

Additional file 1: Data Extraction/Assessment Form (from Hawker et al., [28]). A data extraction form for a Mixed Studies Review methodology. (DOCX 14 kb)

Additional file 2: Modified critical appraisal criteria for empirical studies (from Hawker et al., [28]). A validated critical appraisal tool for empirical studies. (DOCX 15 kb)

## Abbreviations

AMHS: Adult mental health services
CAMHS: Child and adolescent mental health services
PICO: Population, intervention, comparator and outcome
PROMs: Patient-reported outcome measures
