# Supplementary material for: A systematic review of the literature on ethical aspects of transitional care between child- and adult-orientated health services
Source: BMC Med Ethics. 2018 Jul 18;19:73. doi: 10.1186/s12910-018-0276-3 (PMC6052672; doi:10.1186/s12910-018-0276-3)
Supplement: Supplementary file 1 — Data Extraction/Assessment Form (from Hawker et al., [28]). A data extraction form for a Mixed Studies Review methodology. (DOCX 14 kb) [file 12910_2018_276_MOESM1_ESM.docx]

**Appendix 1 Data Extraction/Assessment Form (from Hawker et al., 2002)**

Author(s):

Date of Publication:

Abbreviated Title:

Assessor:

Date Assessed:

Study Design [ ] Quantitative [ ] Qualitative [ ] Combination

Location of Study:

Sample—Description:

Sample—Size:

Aim:

Research Questions/Hypothesis (If Any):

Method and Analysis:

Intervention (If Applicable):

Results:

Conclusions, Comments, and Issues Raised:

|  | Good 4 | Fair 3 | Poor 2 | Very Poor 1 | Comment |
| --- | --- | --- | --- | --- | --- |
| 1. Abstract and title |  |  |  |  |  |
| 2. Introduction and aims |  |  |  |  |  |
| 3. Method and data |  |  |  |  |  |
| 4. Sampling |  |  |  |  |  |
| 5. Data analysis |  |  |  |  |  |
| 6. Ethics and bias |  |  |  |  |  |
| 7. Findings/results |  |  |  |  |  |
| 8. Transferability/ generalizability |  |  |  |  |  |
| 9. Implications and usefulness |  |  |  |  |  |
| Total |  |  |  |  |  |
